# Investigation of Adhesion Properties of Tire—Asphalt Pavement Interface Considering Hydrodynamic Lubrication Action of Water Film on Road Surface

**DOI:** 10.3390/ma15124173

**Published:** 2022-06-12

**Authors:** Binshuang Zheng, Junyao Tang, Jiaying Chen, Runmin Zhao, Xiaoming Huang

**Affiliations:** 1 School of Modern Posts, Nanjing University of Posts and Telecommunications, Nanjing 210023, China; zhengbs@njupt.edu.cn; 2 School of Transportation, Southeast University, Nanjing 211189, China; cjiaying14@seu.edu.cn (J.C.); 230228857@seu.edu.cn (R.Z.); huangxm@seu.edu.cn (X.H.); 3 National Demonstration Center for Experimental Education of Road and Traffic Engineering, Southeast University, Nanjing 211189, China

**Keywords:** tire–pavement contact, texture information identification, numerical modeling, fluid hydrodynamic lubrication theory, orthogonal experimental design, peak adhesion coefficient

## Abstract

To obtain the tire–pavement peak adhesion coefficient under different road states, a field measurement and FE simulation were combined to analyze the tire–pavement adhesion characteristics in this study. According to the identified texture information, the power spectral distribution of the road surface was obtained using the MATLAB Program, and a novel tire hydroplaning FE model coupled with a textured pavement model was established in ABAQUS. Experimental results show that here exists an “anti-skid noncontribution area” for the insulation and lubrication of the water film. Driving at the limit speed of 120 km/h, the critical water film thickness for the three typical asphalt pavements during hydroplaning was as follows: AC pavement, 0.56 mm; SMA pavement, 0.76 mm; OGFC pavement, 1.5 mm. The road state could be divided into four parts dry state, wet sate, lubricated state, and ponding state. Under the dry road state, when the slip rate was around 15%, the adhesion coefficient reached the peak value, i.e., around 11.5% for the wet road state. The peak adhesion coefficient for the different asphalt pavements was in the order OGFC > SMA > AC. This study can provide a theoretical reference for explaining the tire–pavement interactions and improving vehicle brake system performance.

## 1. Introduction

According to the tire–pavement contact mathematical model, the tire optimal slip rate is obtained when the road surface has the peak adhesion coefficient. In addition, the vehicle braking and tire–pavement contact mechanics indicate that the maximum braking deceleration can be achieved during the vehicle braking process under the condition of the peak adhesion coefficient of the road surface [1,2]. Therefore, it is necessary to study the variation characteristics of the asphalt pavement adhesion coefficient for predicting the peak adhesion coefficient of the road surface, which is regarded as the pressure threshold at which the braking system can realize the maximum braking force of the vehicle. Furthermore, the vehicle safety can be effectively improved during the braking process [3]. As one essential component of the tire–pavement friction force, the adhesion force is formed due to the rubber–pavement interaction and is highly dependent on the true area of contact [4,5]. Theoretically, the adhesion results from the shear force on the contact surface of the tire and road. Accordingly, many research institutes have studied the tire–pavement contact model and road safety problem in terms of the tire shear deformation effect, and many typical contact mechanic models between the tire and pavement were summarized in the work of Huang et al. [6]. Recently, a formulation for a deformable solid was constructed using an arbitrary Lagrangian–Eulerian approach [7], and this formulation was applied to a new simplified tire model, represented by a circular ring including shear stresses and nonlinear effects due to the vehicle load. A numerical model of the flexible base asphalt pavement structure under nonuniform vertical tire contact pressure was established, and the effect of nonuniformity and uniformity on the pavement surface deflection was investigated [8]. Yet, no studies have been carried out to investigate the tire–pavement peak adhesion coefficient and its influencing factors.

Related studies have shown that the adhesion of the tire–pavement interface is determined by the macro texture of the road surface [9]. Meanwhile, a water film on the road surface can significantly reduce the tire–pavement adhesion [10,11,12]. Recently, many methods have been proposed to estimate the tire–pavement adhesion characteristics. These method can be divided into two types, cause-based and effect-based. Among them, cause-based methods detect the influencing factors of the adhesion coefficient, such as the road surface texture and fluid lubrication. The estimation method based on the slip rate μ–s curve is typically applied, which mainly considers vehicle acceleration, braking behavior, and lateral movement to estimate the road surface adhesion coefficient. For example, Bachmann [13] proposed the influencing factors of the tire–pavement adhesion coefficient; in particular, the road environmental parameters (road lubricant parameters, such as a water film) had a major impact, but the water film lubrication theory was ignored. German [14] proposed a typical polynomial model to approximate the relationship between the tire–pavement adhesion coefficient and the tire slip ratio using a simple polynomial function. However, this model is inapplicable when the slip ratio is high. The above studies indicated that the tire–pavement adhesion coefficient is closely related to the tire slip rate according to empirical estimation or tire dynamics; however, the adhesion coefficient variation rule has not been revealed from tire–pavement contact theory.

In the previous study, the surface water will generate a certain dynamic water pressure under the pressure of tire load when the vehicle drives on moist asphalt pavement, which can easily cause hydroplaning [15,16]. Most of the water in the tire–road contact area flows or splashes along the surface texture and tire pattern gap. According to Bernoulli theory, it can be known that the micro kinetic energy of the water flow is converted into the pressure of the tire against the road surface [17]. When the tire is running at a certain speed, tire hydroplaning occurs when the elastic hydrodynamic pressure of the water film in the vertical direction of the tire–fluid interface is equal to the tire load.

In theory, the rigidity of the road surface is infinitely flat. Assuming that the tire contact surface is flat, the angle between the tire and the road contact interface is actually very small. It can be considered that the component of the dynamic water pressure in the direction of the tire movement is close to 0. At this time, the vertical component of the hydrodynamic pressure at the stagnation point of the water flow is equal to the total micro kinetic energy, i.e.,

(1) $$G=\int_{s}\frac{\rho_{w}v_{p}^{2}}{2}ds,$$

where *G* is the tire load (kN), *v_p_* is the critical hydroplaning speed of the tire (km/h), *ρ*_w_ is the fluid density (kg/m^3^), and *s* is the action area of the water film (m^2^).

Under the action of the vehicle load, the local elastic deformation of the contact between the tire and the road forms a water film between the lubricated surfaces. The hydrodynamic pressure generated by this water film can be regarded as the elastic deformation of the rubber tread. At this time, the dynamic lubrication state exhibits the phenomenon of elasto–hydrodynamic lubrication (EHL) [18].

Studies have shown that the factors mediating tire hydroplaning under wheel load are tire speed, road conditions (roughness, pavement type, water film thickness, etc.), and tire parameters (pattern structure, rubber material viscoelasticity, tire pressure, and load) [19]. Among them, the road conditions are the main objective influencing factors, and the water film thickness is related to the tire hydroplaning state. On a wet road, a vehicle at high speed can undergo dynamic hydroplaning, viscous hydroplaning, and micro EHL under the influence of the pavement texture and water film. When the water film thickness exceeds 1.0 mm, dynamic hydroplaning often occurs. On rainy days, as it just begins to rain and the road surface becomes wet, a thin water film of 0.01–0.1 mm is formed. According to EHL theory, the water film isolates the contact state between the tire and road surface, resulting in a decrease in the tire–pavement friction. In this case, micro EHL is more likely to occur.

In accordance with fluid hydrodynamic lubrication theory and tire–road contact theory, a tire hydroplaning finite element (FE) model was applied to determine the division of road states considering road surface water film thickness. Luo [20] proposed the limit standard of water film thickness on rainy days considering driving safety using the rainfall data of Hainan region in China, indicating that tire hydroplaning occurs easily when the water film thickness exceeds 4.0 mm. Moreover, Lufft Instrument and Equipment Corporation in Germany developed the advanced mobile road weather information sensor MARWIS, which can test road surface water film thicknesses up to 6 mm. Thus, the water film thickness ranging from 0 mm to 6 mm was taken as the research range to refine the asphalt pavement state.

In the literature, many studies have used the finite element (FE) method [21] and laboratory tests to study tire–pavement contact mechanics [22]. For example, Fwa [23,24] established an FEM for the tire–asphalt pavement interaction, revealing that the change rule of the friction coefficient varied with the thickness of the water film. However, these studies considered the pavement as a smooth flat surface and did not adequately consider the macro and micro texture of the asphalt pavement. On the basis of the tire–pavement contact mechanics, a tire hydroplaning FE model was established, and the tire–pavement adhesion coefficient characteristics were simulated [25,26]. A vehicle braking evaluation method based on the road adhesion coefficient was proposed to evaluate the road adhesion coefficient under the condition of tire sliding in an accident investigation [27]. According to the torque distribution of a single tire, a road adhesion coefficient prediction method was proposed by Ma [28]. Considering road surface fractal theory, Chen [29] tested the stress distribution of tires and found a positive correlation between the average effective stress and the friction coefficient. Guo [30] proposed a method for testing the tire adhesion coefficient and modified the steady–state semi–empirical sidetracking model of the tire. By experimentally examining the relationship between rubber–pavement adhesion and friction, a fair correlation was revealed between the adhesive bond energy and the measured coefficient of friction [5]. As reviewed above, most research results can explain the basic variation rule of the tire–pavement adhesion, but the adhesion characteristics are relative to the road surface fractal theory and rubber friction energy losses.

In view of the above research shortcomings, fluid hydrodynamic lubrication mechanics were applied in this study to demarcate the asphalt pavement states. Then, a field measurement and FE simulation were combined to reveal the tire–pavement adhesion characteristics under different road states. On the basis of the simulation results of the adhesion coefficient, an orthogonal experimental design method was applied to investigate the significant influencing factors for different road states. According to the variation of the simulated adhesion coefficient curves with tire slip rate, peak adhesion coefficient curves were obtained for different driving speeds.

## 2. Materials and Methods

### 2.1. Acquisition of Pavement Texture Information

**Figure 1:**
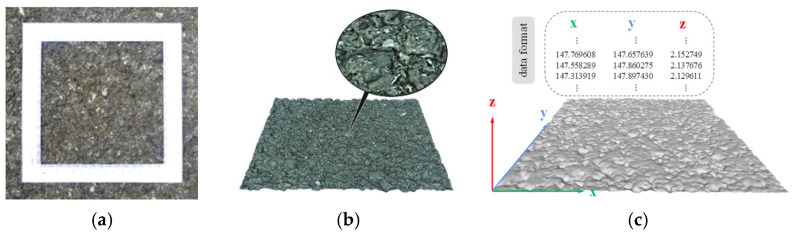
Acquisition of asphalt pavement texture information: (**a**) testing scope of pavement texture; (**b**) 3D digital pavement model; (**c**) 3D data of texture elevation.

**Figure 2:**
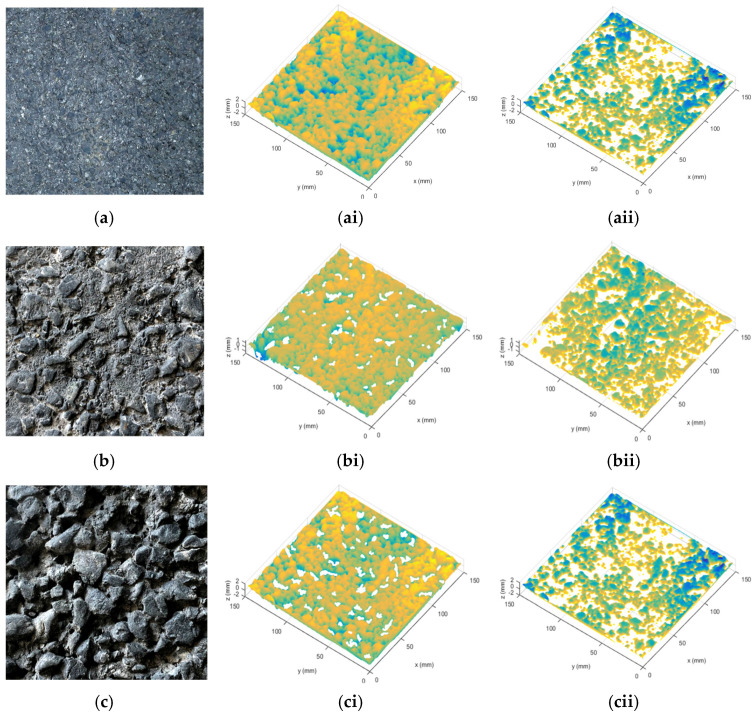
Pavement texture visualization under different conditions: (**a**) AC−13 pavement in (**ai**) dry conditions and (**aii**) wet conditions; (**b**) SMA−13 pavement in (**bi**) dry conditions and (**bii**) wet conditions; (**c**) OGFC−13 pavement in (**ci**) dry conditions and (**cii**) wet conditions. Notes: AC = dense-graded asphalt pavement; SMA = stone matrix asphalt; OGFC = open−grade friction course.

Using the automatic close-range photogrammetry system (ACRP system) developed in [31], the surface texture of three typical asphalt pavements (Figure 1a) was collected. After the preprocessing of the collected images; reverse reconstruction technology was used to reconstruct the three–dimensional (3D) images of the asphalt pavement surface texture, and a 3D model of the asphalt pavement surface texture (Figure 1b) was established. Then, GeoMagic and MeshLab 3D modeling software was adopted to preprocess the reverse reconstructed 3D model of the asphalt pavement surface texture, including hole filling correction, leveling, and definition of local coordinate axis attributes, and the road surface texture 3D elevation data were derived from the 3D model containing (*x*, *y*, *z*) 3D coordinate points (Figure 1c). According to the above 3D elevation data, the surface morphology of the asphalt pavement was reconstructed in MATLAB, as shown in Figure 2.

Three kinds of asphalt pavement were selected in this study: dense–graded asphalt concrete (AC), stone matrix asphalt (SMA), and open–grade friction course (OGFC). The gradation design of the three asphalt mixtures is shown in Table 1. To generate a wet pavement surface, water was uniformly sprayed on the dry surface until the concave asperities were sealed with water [32,33].

**Figure 3:**
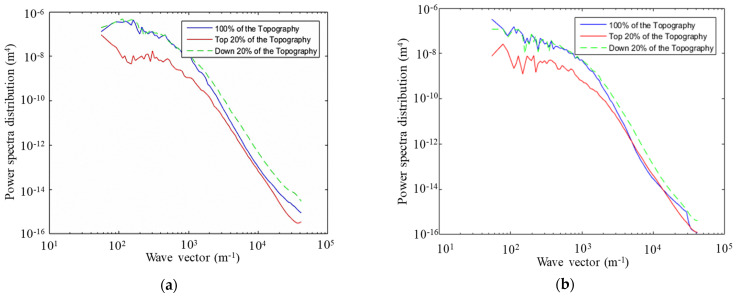

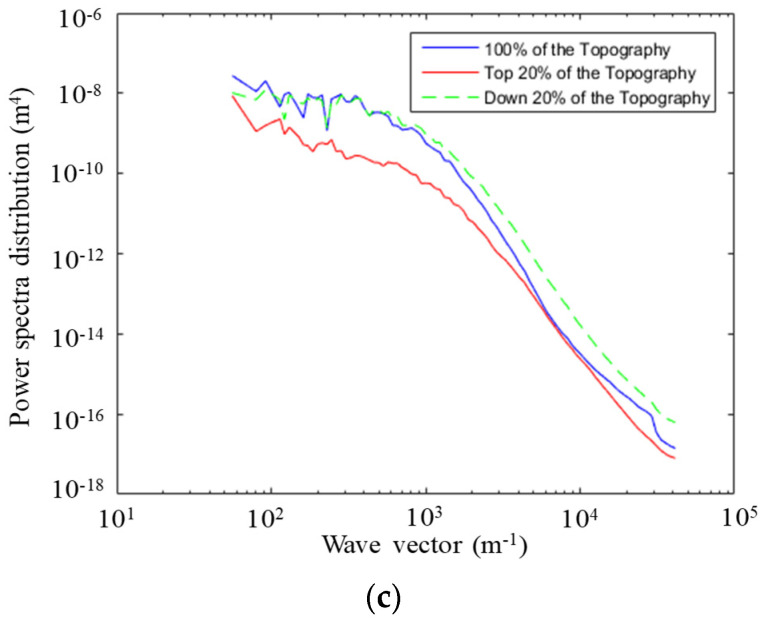
The 2D−PSD of asphalt pavement texture: (**a**) AC−13 asphalt pavement; (**b**) SMA−13 asphalt pavement; (**c**) OGFC−13 asphalt pavement.

On the basis of the obtained 3D texture coordinate elevation data (*x*, *y*, *z*), the power spectral distribution (PSD) solver was written in MATLAB by applying the PSD calculation model for Persson friction theory [34,35]. Since the random variables of the fractal road surface were discrete points, further filtering, windowing, and sampling window compensation of the coordinate data were needed in the process of solving the PSD [19]. The PSD of the road surface under wet and dry conditions was calculated using Equation (2), and the PSD curves for AC−13, SMA−13, and OGFC−13 pavements under different road conditions were obtained as shown in Figure 3.

(2) $$C\left(q\right)=\frac{1}{{\left(2\pi \right)}^{2}}\int \langle h\left(x\right)h\left(0\right)\rangle e^{iqx}dx,$$

where *x* is the wave vector direction, *h*(0) is the surface elevation of the origin point, *h* (*x*) is the surface elevation with the average elevation as the starting point, <…> represents the average on the plane, **q** is the wave vector, which can be obtained through the conversion of wavelength *λ*, and *e* is a natural constant.

**Figure 4:**
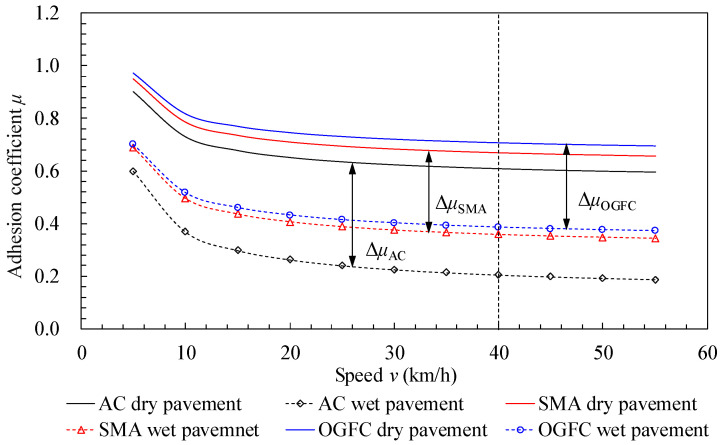
Variation of adhesion coefficient curves with tire speed.

As a function of the calculated PSD, the friction coefficient–velocity curves were obtained using the Persson friction coefficient formula (see Figure 4). The variation trends of the friction coefficient curves under different pavement conditions were similar, both of them decrease significantly with the increase in relative slipping speed.

When the speed exceeded 40 km/h, the curve tended to be gentle, indicating that the actual tire–road contact area was in a steady state at a relatively high speed. The friction coefficient of the wet road surface was lower than that of the dry pavement. In addition, a greater speed resulted in a greater difference in friction coefficient between the two states (dry and wet pavement). Regardless of the road state (dry or wet), the friction coefficient of the different pavements was in the order OGFC > SMA > AC, indicating that the surface roughness did not contribute to skid resistance caused by the water film barrier between the tire and the pavement.

### 2.2. Textured Pavement Modeling in ABAQUS

#### 2.2.1. FE Model of Textured Pavement

For the 3D texture coordinate elevation data, the *.asc format file obtained by the ACRP system was used, containing a set of 3D coordinate points of the road surface, which allowed standardizing the network coordinate points. Furthermore, the .inp file for the 3D digital pavement model was processed in MATLAB and converted into .inp files suitable for the ABAQUS program. The ABAQUS program was adopted to realize the reconstruction of the 3D textured pavement. The specific processing steps for the FE model of the textured asphalt pavement are described below.

(1) Generation of 3D digital pavement model

**Figure 5:**
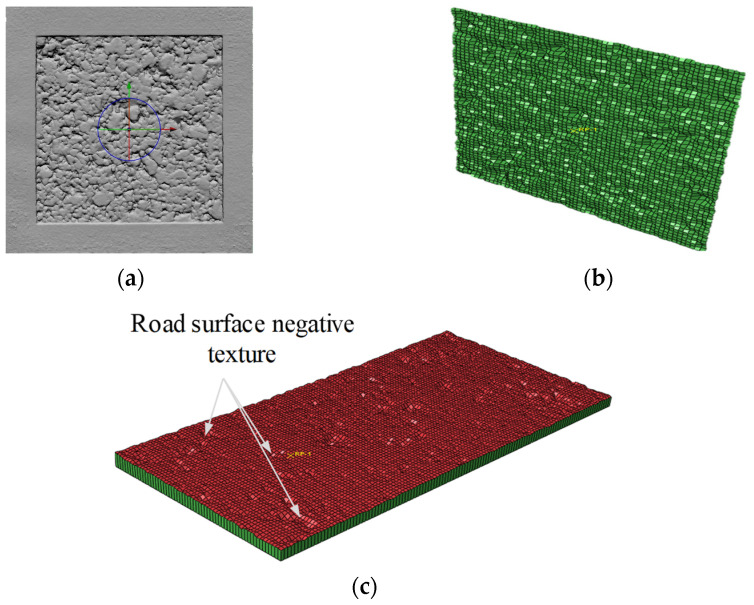
Generation process of textured pavement 3D FE model: (**a**) 3D model of texture morphology; (**b**) rigid shell element; (**c**) textured pavement 3D model.

After image distortion removal and image adjustment, as well as point cloud reconstruction and registration, the 3D digital pavement model was built as shown in Figure 5a. The generated point cloud was matched, converting different sets of point clouds to the same coordinate system using the iterative closest point algorithm. Furthermore, the local axis properties, hole filling correction, and plane leveling were defined.

(2) Unit standardization of grid data

For the generated 3D digital pavement model, the Editplus software was used to extract the 3D texture coordinate elevation data of the .inp file before conversion into .txt format files, which was further realized by the MATLAB program. In MATLAB, the statement “[*m,n*] = *size*” was used to read the array size of the original data file (.txt data file) and generate the same size array storage space. Then, taking the number of data lines as the number of loops, the loop statement was adopted to read the original coordinate points (*x*, *y*, *z*) before unit processing using Equation (3).

(3) $$\{\begin{array}{c} x\left(i,1\right)=\frac{data\left(i,1\right)}{100}\times 1000;\\ y\left(i,1\right)=\frac{data\left(i,2\right)}{100}\times 1000;\\ z\left(i,1\right)=\frac{data\left(i,3\right)}{100}\times 1000. \end{array}$$

(3) Sparsity of grid data

The minimum spacing between the point cloud data was 0.01 mm during the reconstruction process of the point cloud. A smaller spacing results in a denser point cloud, leading to a more realistic reconstructed surface topography. However, the density of the data grid directly exceeded the meshing capability of the ABAQUS preprocessing function, resulting in nonconvergence of the analysis calculation. Through repeated calculations, the minimum unit of the best grid element that allowed convergence was 5 mm. Since the scanned pavement texture data included a square pavement area of 30 × 30 cm^2^, as shown in Section 3.1, the coordinate format of −150:5:150 was used.

After generating the datum coordinate points, the cubic interpolation method based on triangles was used to interpolate the elevation point *zk* through the “*zk = griddata* (*x*, *y*, *z*, *xi*, *yj*, ‘cubic’)” statement. After this interpolation, the sparsity of the road texture coordinate points was generated on the basis of 5 mm plane (*x*, *y*) coordinates. Considering the large difference between the stiffness of the road surface and the stiffness of the tire, the road surface could be regarded as a rigid body in the ABAQUS finite element model. The model of the road rut obtained using the above process was a rigid shell unit. Then, the grid of coordinate points was entered into the preprocessing .inp file in ABAQUS and visualized as shown in Figure 5b.

(4) Extension processing of coordinate data

To provide enough rolling space for the tire in the FE model, it was necessary to expand the distribution characteristics of the pavement texture appropriately. The mirror image processing method was used to establish two mirror images for the coordinate points in step (3) to expand the simulated asphalt road to four times the original size along the tire rolling direction, i.e., 120 cm. This process was realized using the MATLAB “*mirror _ matrix*” statement.

(5) Materialized processing of textured pavement

The rigid shell element cannot restrict the Euler fluid element; that is, the shell element cannot block the water film on the pavement surface. In explicit analysis, solid elements can play a role in blocking water. The digital model of the pavement surface texture generated in step (4) was extended along the *z*–direction by a certain distance to make it a 3D solid unit (in Figure 5c).

#### 2.2.2. Tire Hydroplaning FE Model

**Figure 6:**
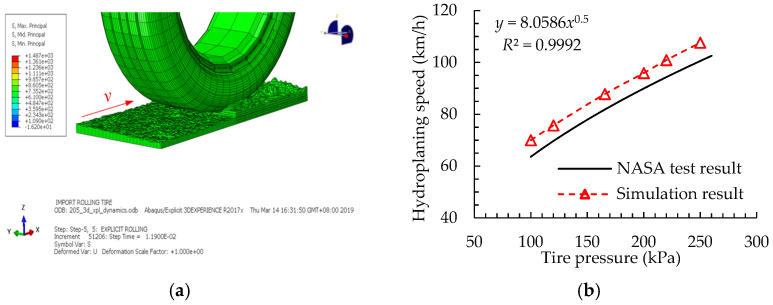
Validation of tire hydroplaning FE model: (**a**) simulation of tire hydroplaning process; (**b**) critical hydroplaning speed of the tire.

The tire hydroplaning model building process in ABAQUS was based on the coupled Euler Lagrange algorithm, as described in [36,37], for which the inflated pattern tire Bridgestone 205–55–R16 was adopted [25]. Then, the dynamic friction coefficient curves (in Figure 6) between the tire and pavement under different road conditions were imported into the built hydroplaning model.

The critical hydroplaning speed of the tire calculated using the National Aeronautics and Space Administration (NASA) empirical formula was taken as the initial speed [38]. Then, the critical hydroplaning speed under different inflatable pressures was simulated by adjusting the rolling speed of the tires according to the tire hydroplaning FE model, as shown in Figure 6b. From the variation curve of the tire hydroplaning speed, it can be seen that, as the tire inflation pressure increased, the critical hydroplaning speed of the tire gradually increased. Moreover, it changed linearly with the square root of the tire pressure. According to the regression analysis, the predictive formula of the tire hydroplaning speed was obtained as *v*_crit_ ≈ 8.06*P*^0.5^, which is consistent with the NASA test results.

Nevertheless, the fitting coefficient of the formula was slightly higher than that of the NASA empirical formula, which results from the difference in tire pattern parameters and road surface texture characteristics. However, the simulated critical hydroplaning speed was within the allowable range of error, verifying the accuracy of the hydroplaning FE model.

For the proposed hydroplaning model, Equation (4) was used to calculate the tire adhesion coefficient under wet conditions. In the model, the tire rolled in the *z*–direction; hence, the rolling resistance of the tire was calculated as a function of the joining force in the *z*–direction received by the reference point on the rim.

(4) $$\mu_{s}=\frac{\mu \left(F_{h}-F_{t}\right)+F_{d}}{F_{h}}=\frac{F_{z}}{F_{h}},$$

where *µ_s_* is the tire adhesion coefficient under wet conditions, *µ* is the tire adhesion coefficient under dry conditions, *F_d_* is the fluid drag force, *F_h_* is the tire axle load, *F_z_* is the tire rolling resistance, and *F_t_* is the fluid lift force.

On a dry road, the adhesion coefficient is the tangential force divided by the normal load. The tire−pavement contact area contains the adhesion region and the slipping region. The tangential reaction force on the tire is the sum of the longitudinal force in the adhesion region and the sliding friction in the slipping region [39]. Hence, the adhesion coefficient can be calculated as follows:

(5) $$\mu =\frac{F_{xn}+F_{xb}}{F_{h}}<\mu_{f},$$

where μ is the adhesion coefficient under dry conditions, $\mu_{f}$ is the slipping friction coefficient, $F_{xn}$ is the slipping friction in the adhesion region, and $F_{xb}$ is the slipping friction in the slipping region.

## 3. Results

### 3.1. Pavement State Demarcation

**Figure 7:**
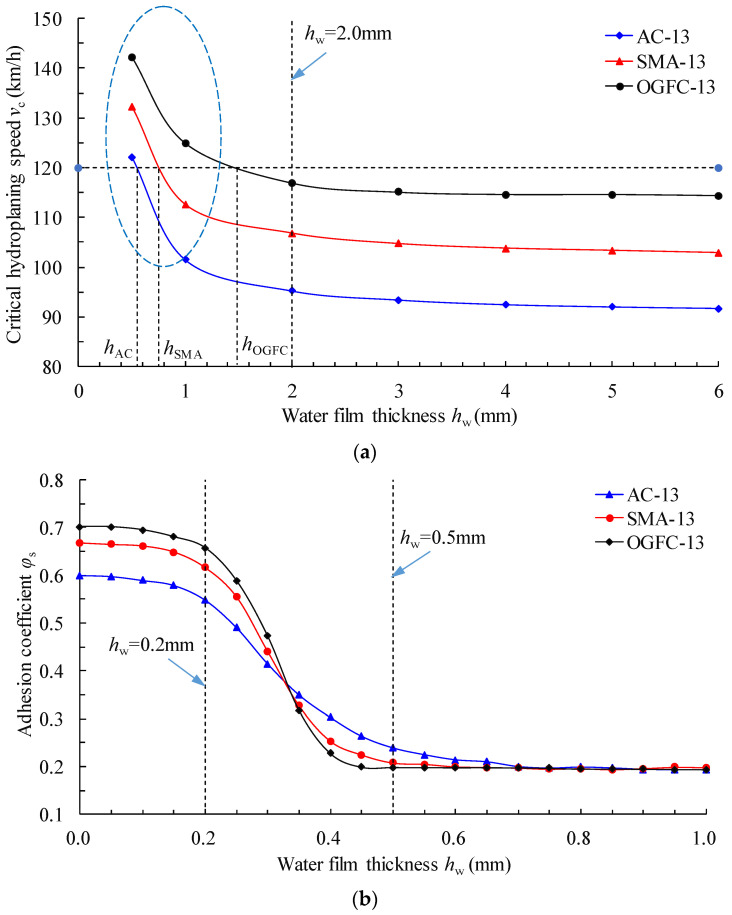
Influence of water film thickness on tire–pavement contact characteristics: (**a**) critical hydroplaning speed of the tire; (**b**) adhesion coefficient between tire and pavement.

According to the established tire hydroplaning FE model, the tire load of 3.922 kN and the inflation internal pressure of 250 kPa remained unchanged. The influence of the water film thickness on the critical hydroplaning speed for different asphalt pavements was analyzed. Considering the actual vehicle hydroplaning risk, the vehicle was deemed to already be in the hydroplaning state within the limit speed when the water film thickness was greater than 6 mm. Thus, the water film thickness within the range of 0–6 mm was considered. According to the definition of tire hydroplaning, the minimum driving speed is the critical hydroplaning speed when the tire–pavement contact force is equal to 0. The critical hydroplaning speed for different water film thicknesses was obtained as shown in Figure 7a.

Within a certain range of water film thickness, the trends of the critical hydroplaning speeds of the three typical asphalt pavements were generally consistent (Figure 7a). When the thickness of the water film was in the range of 0–2 mm, the critical hydroplaning speed of tire change rate was significant. When the water film thickness was greater than 2 mm, the critical hydroplaning speed curve tended to be stable. Driving at the specified speed limit of 120 km/h, the critical water film thickness *h*_crit_ for the three typical asphalt pavements for which tire hydroplaning occurred was as follows: AC, 0.56 mm; SMA, 0.76 mm; OGFC, 1.5 mm. This is mainly because the macro texture of the road surface provided a skid resistance force. In this case, the friction provided by the micro texture of the road surface was minimal. Obviously, when the water film thickness on the road surface was less than the critical value *h*_crit_ (0 < *h*_w_ < *h*_crit_), the vehicle was not at risk of hydroplaning. Research results showed that the pavement conditions could be defined as the wet state when the water film thickness met the condition of 0 < *h*_w_ < *h*_crit_. Considering that the pattern depth limit of car tires is 1.6 mm, and that the highway speed limit is 120 km/h, the critical water film thickness *h*_crit_ was calculated to be 1.021 mm using Equation (6) [40,41]. Therefore, the wet state of the road surface could be defined as a water film thickness of 0 < *h*_w_ ≤ 1 mm.

(6) $$v_{p}=v_{\mathrm{crit}}+12\frac{t}{h}+60\exp\left\{-3\left[h-\left(3+\frac{t}{b}\right)\right]\right\},$$

where *v*_crit_ is the critical hydroplaning speed of the tire (*v*_crit_ = 6.35*p*^1/2^), *p* is the tire inflation pressure (kPa), *t* is the tread pattern depth (mm), *h* is the water film thickness on the road surface (mm), and *b* is the tread width of the tire (mm).

According to the tire–pavement contact mechanism, there is a minimum skid resistance of the road surface before the critical water film thickness appears. In this case, the micro convex body on the road surface is completely wrapped by the water film, and the frictional resistance suffered by the tire is almost entirely derived from the viscous force of the lubricating medium (water). This critical state is called the dividing line between the wet state and ponding state, at which the skid resistance of the road surface is the worst. In view of this, it is necessary to refine the adhesion characteristics of the tire–fluid–road surface with a water film thickness of less than 1 mm from the micro contact theory.

Setting the initial speed of the tire to 60 km/h and keeping the other parameters unchanged, the water film thickness in the established hydroplaning FE model was adjusted. The influence of the water film thickness within the range of 0–1 mm on the adhesion coefficient was discretized. The variation of the adhesion coefficient curves with the water film thickness is shown in Figure 7b. It can be seen that the adhesion coefficient curves could be divided into three phases:

- Boundary lubrication stage (the water film thickness ranges from 0 to 0.2 mm). Here, the fluid lubrication effect was very small, but the road surface micro convex body had a large contribution rate. The friction between the tire and pavement depended on the asperity of the road surface.
- Mixed lubrication stage (the water film thickness ranges from 0.2 mm to 0.5 mm). Part of the road surface micro convex body was blocked by the water film and became an “anti−skid noncontributing area”. At this time, the friction characteristics of the road surface were determined by the fluid viscosity and surface roughness.
- Elastic fluid lubrication stage (the water film thickness ranges from 0.5 mm to 1.0 mm). At this stage, the road surface micro convex body was completely submerged by the water film. However, the road surface water film thickness was low, and no dynamic water pressure was generated.

According to the theory of elastic hydrodynamic lubrication, the pavement could be divided into four states considering the water film thickness and the corresponding sources of the adhesion coefficient:

- In the dry state, i.e., *h*_w_ = 0 mm, the adhesion coefficient between the tire and pavement mainly depended on the texture characteristics of the road surface contact surface.
- In the wet state or moist state, i.e., 0 < *h*_w_ = 0.5 mm, the adhesion coefficient was determined by the roughness of the road surface and the fluid viscosity.
- In the lubrication state, i.e., 0.5 mm < *h*_w_ ≤ 1 mm, the adhesion coefficient was negligible, the adhesion force depended on the fluid viscosity resistance, and the tire slip rate increased rapidly.
- In the ponding state, i.e., *h*_w_ > 1 mm, the adhesion force of the tire depended entirely on the fluid viscosity resistance. At this time, there was a risk of tire hydroplaning.

According to the above analysis, different road states had different sources of tire–pavement adhesion coefficients, and the corresponding adhesion coefficient could be determined by the specific road surface type, water film thickness, vehicle speed, and tire inflation pressure. In a dry road state, the adhesion coefficient is mainly determined by the roughness of the road surface. On a wet road, the “anti–skid noncontributing area” of the road surface should be considered.

### 3.2. Influencing Factors of Adhesion Coefficient

#### 3.2.1. In a Dry Road State

**Figure 8:**
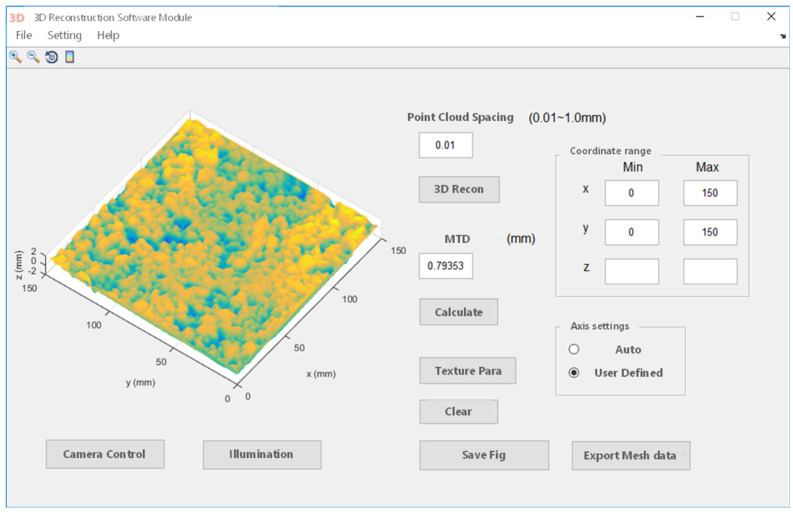
The 3D reconstruction software module.

Through the ACRP system, three kinds of asphalt pavement texture information were acquired. The MATLAB program was applied to write a code for realizing 3D coordinate point data import and visualization of the asphalt pavement surface texture. Then, the mean texture depth (MTD) values were calculated; the visual interface is shown in Figure 8. Compared with the sand patch method, the results show that the errors were all within 5%, further verifying the accuracy of the MTD value obtained through the ACRP system, as shown in Table 2.

**Figure 9:**
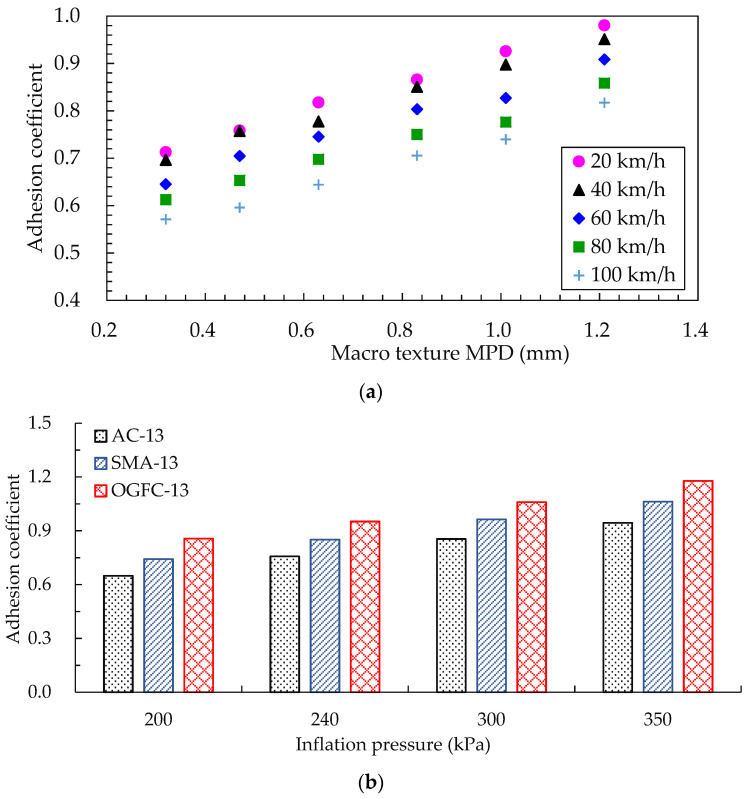
Influencing factors of adhesion coefficient in a dry road state: (**a**) influence of macro texture on adhesion coefficient; (**b**) influence of inflation pressure on adhesion coefficient.

The tire internal pressure was set to 240 kPa, and the load was set to 3.922 kN. According to the test data in Table 2, the MPD values of the road surface macro texture of 0.32 mm, 0.47 mm, 0.63 mm, 0.83 mm, 1.01 mm, and 1.21 mm were chosen to analyze the variation of the road surface adhesion coefficient under different driving speeds, as shown in Figure 9a. As the MPD value gradually increased, the adhesion coefficient between the tire and the road increased, and the increasing trend was more significant at higher driving speeds. For example, when the driving speed was 40 km/h, the adhesion coefficient increased by 33.7%, whereas it increased by 47.1% at a speed of 100 km/h. Apparently, the macro texture of the pavement helped to improve the adhesion of the pavement.

The tire speed was set to 60 km/h, and the load was set to 3.922 kN; then, the tire inflation pressure was adjusted in the established hydroplaning FE model. The influence of tire inflation pressure on the adhesion coefficient was investigated, and the results are shown in Figure 9b. It can be seen that the adhesion coefficient between the tire and the road surface increased with the increase in tire inflation pressure; the adhesion coefficient curves were distributed as a parabola, and the rate of increase decreased with the increase in the inflation pressure. Comparing the change trend of the adhesion coefficient of three typical asphalt pavements, it can be found that the OGFC asphalt pavement had the largest adhesion coefficient under the same tire inflation pressure, followed by SMA and AC asphalt pavement. A greater inflation pressure resulted in a greater change rate of the OGFC asphalt pavement adhesion coefficient.

#### 3.2.2. Under Wet Road Conditions

**Figure 10:**
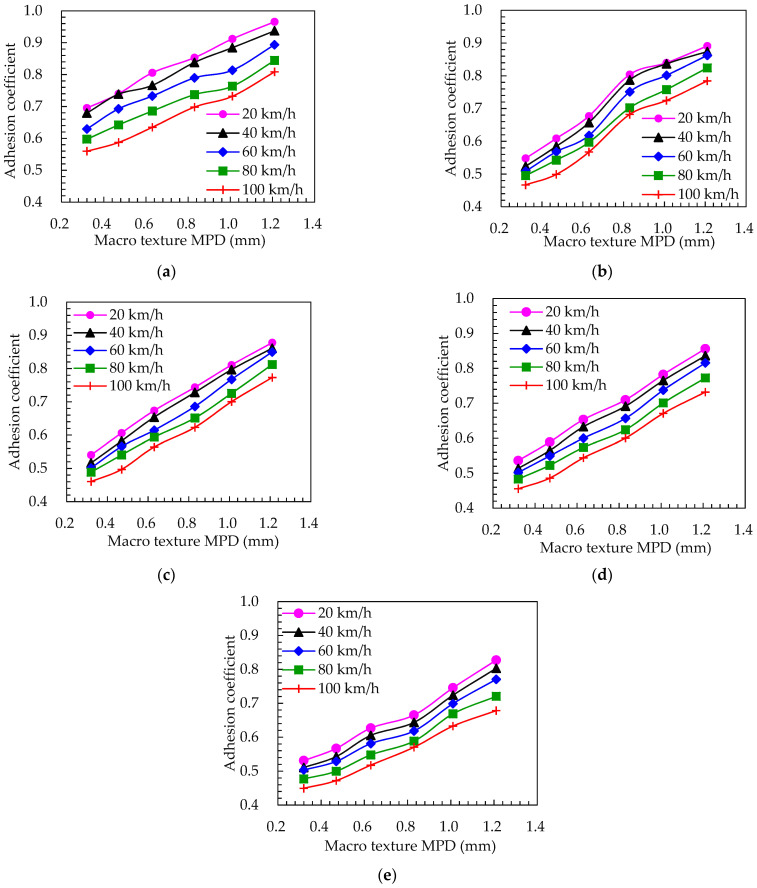
(**a**) *h*_w_ = 0.2 mm; (**b**) *h*_w_ = 1.0 mm; (**c**) *h*_w_ = 2.0 mm; (**d**) *h*_w_ = 3.5 mm; (**e**) *h*_w_ = 5.0 mm. Influence of water film thickness on adhesion coefficient in a wet road state.

The calculation of the road surface adhesion coefficient under different water film thicknesses was simulated using Equation (4). Furthermore, the influence of the speed and the macro texture of the road surface on the adhesion coefficient between the tire and the road surface was analyzed. The adhesion coefficient curves under different water film thicknesses were obtained as shown in Figure 10. In addition, the variable values of water film thickness covered all road states.

For certain macro texture parameters and rolling speeds, the road surface adhesion coefficient gradually decreased as the water film thickness increased. When the water film thickness was *h*_w_ ≤ 1.0 mm, the pavement adhesion coefficient was larger and the change rate of the adhesion coefficient was higher as the macro texture increased. When the water film thickness was *h*_w_ > 1.0 mm, the adhesion coefficient gradually decreased. Moreover, the influence degree of the road surface macro texture decreased rapidly, indicating that the road surface adhesion characteristics mainly depended on the viscosity of the water flow when the water film thickness was greater than 1 mm (in a moist road state).

## 4. Discussion

### 4.1. Sensitivity Analysis Based on Orthogonal Experimental Design

#### 4.1.1. In a Dry Road State

Based on the simulation result of the adhesion coefficient in Section 3.2.1, the adhesion coefficient between the tire and pavement was taken as the objective function, the macro texture MPD, speed, and tire inflation pressure were considered as test factors in a dry road state (Table 3). An orthogonal table L_16_(4^5^) is used to represent orthogonal experiment with five factors and four levels was selected to arrange the orthogonal test. Here, *k*_1_, *k*_2_, *k*_3_, *k*_4_, and *k*_5_ represented the MPD value, tire speed, inflation pressure, and two blank columns, respectively. The analysis results of the orthogonal experimental design are shown in Table 4.

As shown in Table 3, the factors were arranged from significant to insignificant as follows: MPD value, speed, and tire inflation pressure. Moreover, the optimal combination of factors was *k*_1_*x*_1_, *k*_2_*x*_4_, and *k*_3_*x*_2_, and the most disadvantageous combination was *k*_1_*x*_4_, *k*_2_*x*_1_, and *k*_3_*x*_4_. According to variance analysis results, the significance level of each factor was consistent with the range analysis results. As shown in Table 4, the macro texture MPD value of the road surface was the most significant factor, whereas the influence of speed and tire inflation pressure could be neglected.

#### 4.1.2. In a Wet Road State

Similarly, the adhesion coefficient between the tire and pavement was taken as the objective function in a wet road state according to the simulation results in Section 3.2.2. Furthermore, the water film thickness, macro texture MPD, and tire speed were considered as the test factors (Table 5). An orthogonal table L_25_(5^6^) representing an orthogonal experiment with six factors and five levels was selected to arrange the orthogonal test. Here, *k*_1_, *k*_2_, *k*_3_, *k*_4_, *k*_5_, and *k*_6_ represented the water film thickness, macro texture MPD, tire speed, and three blank columns, respectively. The analysis results of the orthogonal experimental design are shown in Table 6.

According to the results of orthogonal experimental, it can be seen that the macro texture of road surface was the most significant factor, followed by water film thickness and tire speed, which is consistent with the results in a dry road state. Whether in a dry state or wet state, the macro texture parameter of the road surface was the most significant factor. Thus, the three typical kinds of asphalt pavement were selected as the macro texture parameter variables to analyze the change rule of the peak adhesion coefficient.

### 4.2. Peak Adhesion Coefficient for Different Road States

#### 4.2.1. In a Dry Road State

**Figure 11:**
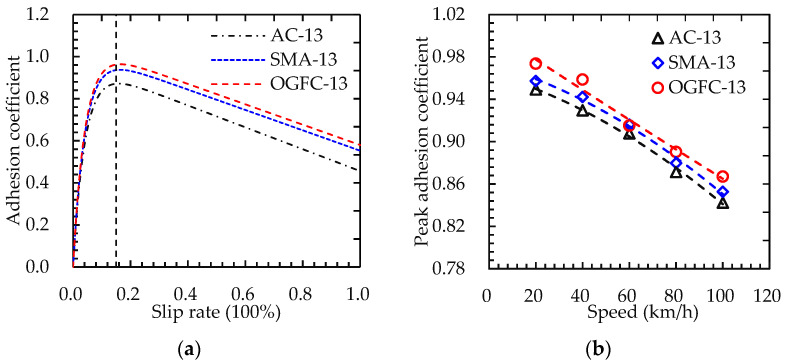
Adhesion characteristics of asphalt pavement in a dry road state: (**a**) variation of adhesion coefficient with slip rate; (**b**) peak adhesion coefficient.

Considering the significant influencing factors of the adhesion coefficient, only the adhesion coefficient variation of different asphalt pavement types with tire slip rates was considered in this study. The test speed of the tire was defined as 60 km/h, and the simulation results are shown in Figure 11a. When the slip rate was around 15%, the adhesion coefficient reached the maximum value, indicating the peak adhesion coefficient.

The tire slip rate was maintained in the optimal range (around 15%), and the rolling speed of the tire model was adjusted while keeping other parameters constant. Then, the peak adhesion coefficient curves for different asphalt pavements were obtained as shown in Figure 11b. The peak adhesion coefficient of the road surface gradually decreased with the increase of vehicle speed, and the peak adhesion coefficient of the road surface was distributed as a convex parabola. This is mainly because the tire rolling radius became large at high speed, resulting in a greater contact area between tire and road surface. Thus, the adhesion force provided by the road surface was reduced.

#### 4.2.2. In a Wet Road State

**Figure 12:**
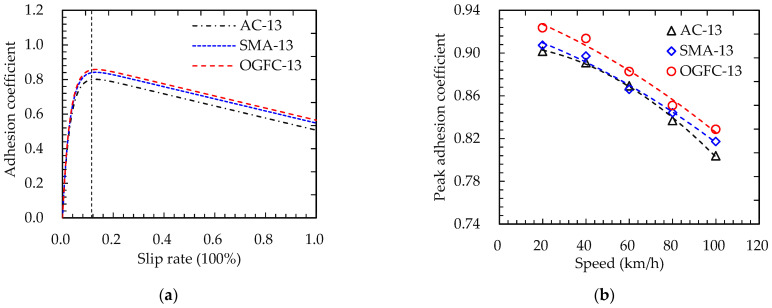
Peak adhesion coefficient curves of asphalt pavement: (**a**) adhesion coefficient varied with slip rate; (**b**) peak adhesion coefficient.

Considering the influencing factors of adhesion characteristics under wet road conditions analyzed in Section 3.2.2, it can be seen that the adhesion coefficient in a wet state (0 < *h*_w_ ≤ 0.5 mm) was the largest. With the accumulation of rainfall and time, the thickness of the surface water film increases gradually. Under the effect of water lubrication, the road surface texture is “sealed” and cannot provide frictional resistance. The adhesion force between the tire and the road surface mainly results from the fluid viscous force. Thus, the adhesion coefficient of the road surface in a typical wet road state (*h*_w_ = 0.2 mm) was taken as research object. The influence of macroscopic texture parameters on the peak adhesion coefficient of the road surface was analyzed. Three typical kinds of asphalt pavement characterizing different macro texture intervals were selected to obtain the variation of adhesion coefficient curves with the tire slip rate, as shown in Figure 12a. With an increase in the tire slip rate, the adhesion coefficient between the tire and pavement quickly increased and then gradually decreased. When the slip rate was around 11.5%, the adhesion coefficient curves attained their peak.

Similarly, the tire slip rate was maintained in the optimal range (around 11.5%), and the rolling speed of the tire model was adjusted while keeping other parameters constant. According to the simulation results, the peak adhesion coefficient curves for three kinds of asphalt pavement under different vehicle speeds were obtained, as shown in Figure 12b.

It can be seen that the peak adhesion coefficients for all three kinds of asphalt pavement at the same vehicle speed had the following relationship in a wet road state: OGFC pavement > SMA pavement > AC pavement. Moreover, the peak adhesion coefficient on OGFC pavement in a dry road state was larger than that on AC pavement in a wet road state. Apparently, the peak adhesion coefficient in a wet road state was slightly lower than that in a dry road state, which was mainly determined by the contribution rate of the pavement texture.

## 5. Conclusions

In this study, an automatic close-range photogrammetry system was adopted to acquire asphalt pavement texture information. Then, the road power spectrum density curve was calculated using MATLAB according to Persson friction theory. Using the established tire hydroplaning model, the influence of water film thickness on the tire–pavement contact characteristics was analyzed, and the road states were demarcated according to fluid hydrodynamic lubrication theory. For different road states, the significant influencing factors of the tire–pavement adhesion characteristics were investigated using an orthogonal experimental design, and the peak adhesion coefficient was calculated. The main results are presented below.

1. For both dry and wet road conditions, the adhesion coefficient of different types of pavements could be ordered as OGFC pavement > SMA pavement > AC pavement. The change rules of the adhesion coefficient curves under different pavement conditions were basically similar, both decreasing significantly with the increase in relative slipping speed. When the speed exceeded 40 km/h, the curves tended to be gentle. For the insulation and lubrication of the water film, there existed an “anti-skid noncontribution area”.
2. When the thickness of the water film was in the range of 0–2 mm, the critical hydroplaning speed of tire change rate was significant. When the water film thickness was greater than 2 mm, the critical hydroplaning speed curve tended to be stable. When driving at the specified speed limit of 120 km/h, the critical water film thickness for the three typical asphalt pavements that resulted in hydroplaning was as follows: AC, 0.56 mm; SMA, 0.76 mm; OGFC, 1.5 mm. According to the theory of elastic hydrodynamic lubrication, the pavement could be divided into four states: dry state, wet sate, lubricated state, and ponding state.
3. In a dry road state, as the MPD value gradually increased, the adhesion coefficient between the tire and the road increased, and the increasing trend was more significant at higher driving speeds. Upon increasing the tire inflation pressure, the adhesion coefficient increased and was distributed as a parabola. The OGFC asphalt pavement had the largest adhesion coefficient under the same tire inflation pressure, followed by SMA and AC asphalt pavement. A greater inflation pressure led to a greater change rate of the adhesion coefficient for the OGFC asphalt pavement.
4. In a wet road state, for certain macro texture parameters and rolling speed, the road surface adhesion coefficient gradually decreased as the water film thickness increased. When the water film thickness was *h*_w_ ≤ 1.0 mm, the pavement adhesion coefficient was larger and the change rate of the adhesion coefficient was higher as the macro texture increased. When the water film thickness was *h*_w_ > 1.0 mm, the adhesion coefficient gradually decreased.
5. According to the sensitivity analysis results, whether in a dry state or wet state, the macro texture parameter of the road surface was the significant factor. In a dry road state, when the slip rate was around 15%, the adhesion coefficient reached the peak value, i.e., around 11.5% for the wet road state. At the same speed, the peak adhesion coefficient for different asphalt pavements could be ordered as OGFC pavement > SMA pavement > AC pavement for both the dry state and the wet state. Moreover, the peak adhesion coefficient on OGFC pavement in a dry road state was larger than that on AC pavement in a wet road state.

In this paper, a field measurement and FE simulation were combined to reveal the tire–pavement adhesion characteristics under different road states, which can provide a comprehensive information for explaining the tire–pavement interaction. As a function of the different road surface peak adhesion coefficients *μ*_max_ obtained, the desired braking deceleration *a_des_* of autonomous vehicles can be calculated using the formula *a_des =_* *μ*_max_ *g*. Thus, the research results in this paper can provide key parameters for further research on autonomous braking performance.

## Figures and Tables

**Table 1:** Gradations for three kinds of asphalt pavement.

	Sieve Size (mm)	Passing Rate of Each Sieve (%)									
Components		0.075	0.15	0.3	0.6	1.18	2.36	4.75	9.5	13.2	16
AC−13		6	10	13.5	19	26.5	37	53	76.5	95	100
SMA−13		10	13.2	16.3	19.5	22.7	25.8	29	63.5	97.9	100
OGFC−13		4.6	5.4	6.1	8.7	11.5	15.0	18.8	63.3	97.8	100

**Table 2:** The MTD values for three kinds of asphalt pavement.

Asphalt Pavement Type	Specimen	Sand Patch Method (mm)	ACRP System (mm)	Relative Error (%)
AC−13	1	0.45	0.44	0.16
	2	0.48	0.48	0.37
	3	0.49	0.49	0.47
SMA−13	1	0.61	0.61	1.01
	2	0.75	0.74	0.71
	3	0.84	0.84	1.20
OGFC−13	1	0.86	0.85	0.81
	2	0.92	0.91	0.80
	3	0.93	0.93	0.48

**Table 3:** Levels of influence factors in a dry road state.

No.	MPD Value (mm) *k*_1_	Tire Speed (km/h) *k*_2_	Tire Inflation Pressure (MPa) *k*_3_
1	0.47	40	200
2	0.63	60	240
3	0.83	80	300
4	1.01	100	350

**Table 4:** Range and variance analysis of factors in a dry road state.

Analysis Item	Test Factors				
	*k*_1_	*k*_2_	*k*_3_	*k*_4_	*k*_5_
*x*_1_	0.670	0.813	0.742	0.740	0.745
*x*_2_	0.716	0.778	0.741	0.749	0.748
*x*_3_	0.785	0.719	0.744	0.751	0.751
*x*_4_	0.810	0.671	0.756	0.742	0.738
*R*	0.140	0.142	0.015	0.011	0.013
*df*	3	3	3	3	3
*SS*	0.049	0.047	0.001	0.0014	0.0016
*F*	2.526	2.423	0.052	—	—
*F*_crit_ 0.10	2.490	2.490	2.490	—	—
Significance level	Significant	Insignificant	Insignificant	—	—

**Table 5:** Levels of influence factors in a wet road state.

No.	Water Film Thickness (mm) *k*_1_	MPD Value (mm) *k*_2_	Tire Speed (km/h) *k*_3_
1	0	0.32	20
2	0.2	0.47	40
3	1.0	0.63	60
4	2.0	0.83	80
5	5.0	1.01	100

**Table 6:** Range and variance analysis of factors in a wet road state.

Analysis Item	Test Factors					
	*k*_1_	*k*_2_	*k*_3_	*k*_4_	*k*_5_	*k*_6_
*x*_1_	0.741	0.566	0.722	0.665	0.662	0.673
*x*_2_	0.733	0.611	0.714	0.668	0.660	0.659
*x*_3_	0.630	0.655	0.665	0.667	0.675	0.677
*x*_4_	0.631	0.708	0.627	0.673	0.666	0.653
*x*_5_	0.582	0.777	0.590	0.645	0.654	0.650
*R*	0.159	0.211	0.132	0.028	0.021	0.027
*df*	4	4	4	4	4	4
*SS*	0.099	0.136	0.063	0.00074	0.00055	0.00071
*F*	1.954	2.684	1.243	1	1	1
*F*_crit_ 0.10	2.190	2.190	2.190	—	—	—
Significance level	Insignificant	Significant	Insignificant	—	—	—

## Data Availability

The data used to support the findings of this study are available from the first author upon request. In this paper, some models and codes used during the study are proprietary or confidential in nature and may only be provided with restrictions, such as the PSD calculation code foe MATLAB software and the subroutine of the tire–pavement interface in ABAQUS.
